# A novel approach to determine the surface area of buckspoor spider webs and other irregular-shaped two-dimensional objects

**DOI:** 10.1016/j.mex.2022.101904

**Published:** 2022-11-02

**Authors:** Christiaan Venter, Charles Richard Haddad, Daryl Codron

**Affiliations:** a Department of Mathematics and Applied Mathematics, University of the Free State, P.O. Box 339, Bloemfontein 9300, South Africa; b Department of Zoology and Entomology, University of the Free State, P.O. Box 339, Bloemfontein 9300, South Africa

**Keywords:** Burrow, Nama Karoo, Peripheral length, Pixels, Silk

## Abstract

Arising from the practical need to determine the surface area of the buckspoor spider webs of *Seothyra schreineri*, an approach for area determination was developed based on image processing. The method rendered results that were considered valid and reliable. Aside from the present application to determine the surface area of a spider web, this approach could potentially be used to accurately determine the area of any two-dimensional shape. For each web's photograph:

• The boundary of the web was outlined.
• Seeded region growing was used to segment the image into a web region and a background region.
• The area of the web was determined as a pixel count, and then converted to mm^2^.

## Graphical abstract

**Figure:**
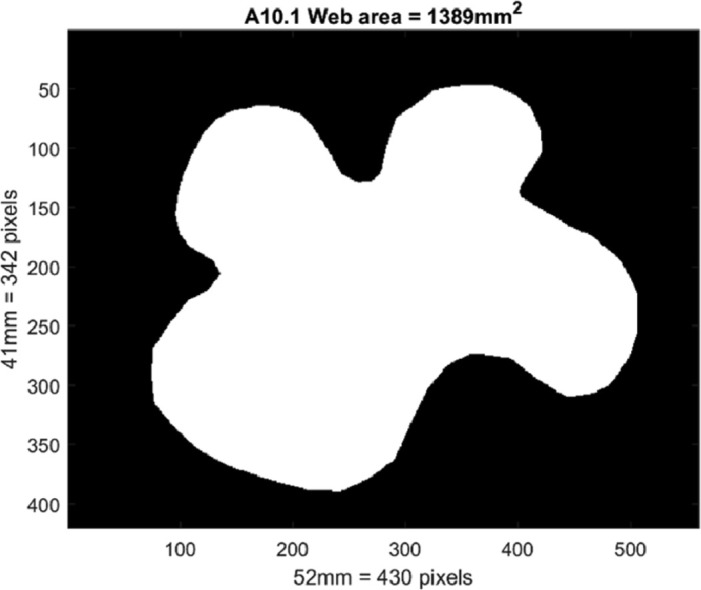

Specifications table

**Table:** 

Subject area	Agricultural and Biological Sciences
More specific subject area	Arachnology
Name of your method	A novel approach to determine the surface area of buckspoor spider webs and other irregular-shaped two-dimensional objects
Name and reference of original method	Not applicable
Resource availability	CorelDraw X7, GNU Octave 7.1.0

## Introduction

Spiders are a mega-diverse arthropod order, with more than 50 000 species worldwide [1]. Approximately a third of the species construct silk webs with which prey is captured, while the majority of species hunt their prey. One such group is the velvet spiders (Eresidae), a small group of approximately 102 species that mostly construct an asymmetrical retreat web on the soil, under rocks or under bark [2]. Only the genus *Stegodyphus*, comprising social and solitary species, constructs a retreat web in the foliage of plants [3,4].

**Fig. 1:**
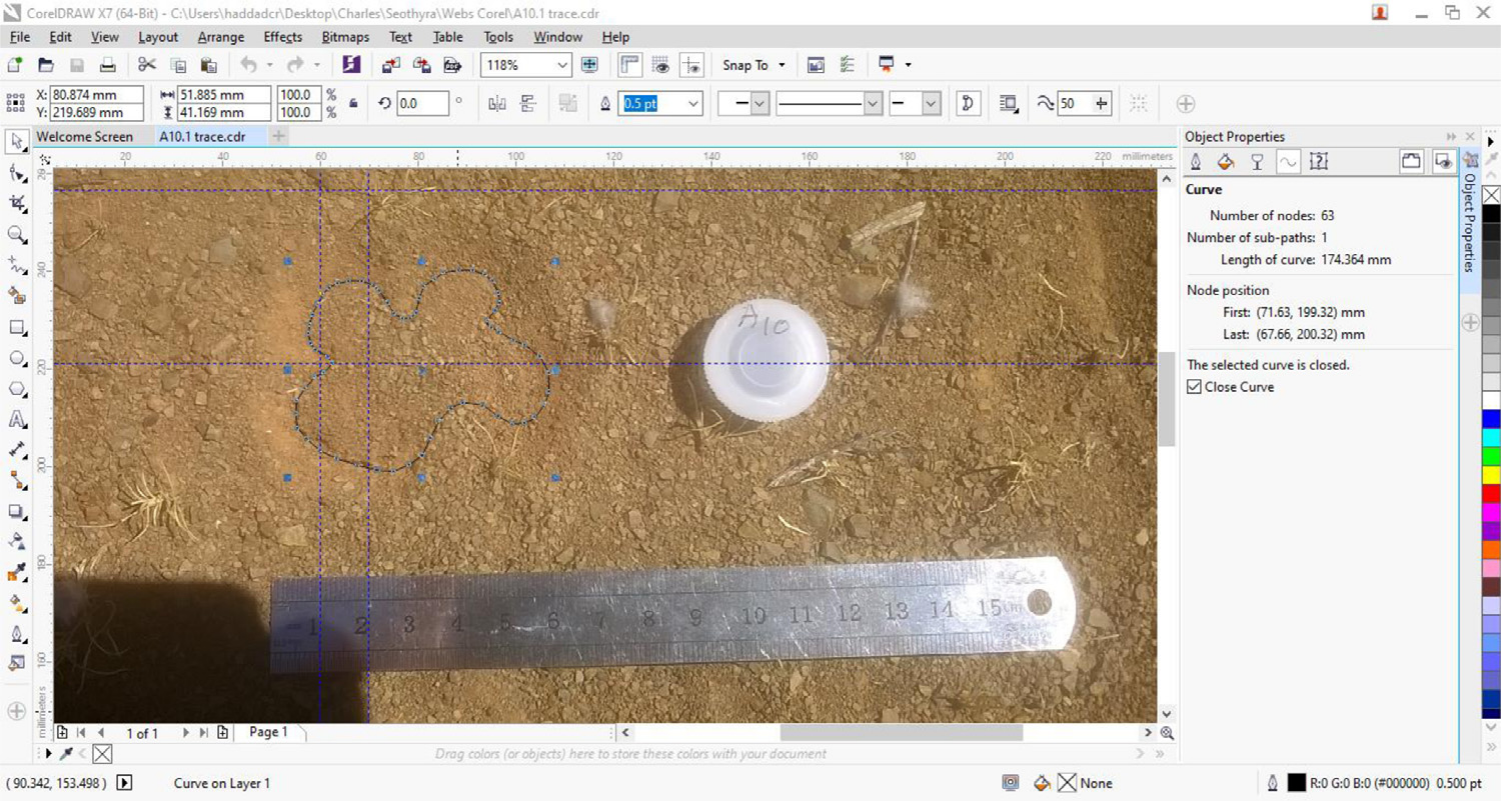
Screenshot of the initial processing of a *Seothyra schreineri* web in CorelDraw X7, with web A10.1 as a representative example, demonstrating the image resizing to scale using a steel ruler as a measure, the trace constructed of the web periphery using the Bezier tool, and the object properties (including peripheral length, 174.364 mm). The distance between the two vertical blue lines is 10 mm. Copyright © 2022_Charles Haddad, Corel Corporation and its licensors. All rights reserved.

The genus *Seothyra* is peculiar among spiders, because it constructs a burrow in the soil, with a silk web flush with the soil surface that resembles a four-leaf clover or antelope hoof print (“spoor”) (Fig. 1), hence their common name, buckspoor spiders. The general shape of the webs consists of four lobes in adults, but this may be reduced to three or two lobes in immature spiders, with deep bays between each pair of lobes. The webs essentially consist of a sheet of densely woven cribellate silk and are covered in sand and other debris from the surrounding substrate. This makes most of the web ineffective for prey capture, as only the peripheral sticky silk can effectively trap arthropod prey [5,6], with the spider rushing from the burrow to the surface to capture the trapped organism and drag it beneath the web [7], [8], [9].

Considering the irregular shaped, asymmetrical form of a buckspoor web, calculating the surface area can be extremely challenging, and may lead to inaccurate estimations. Here we present a method to first trace the outline of a web to scale, and then fill the traced object with digital pixels to determine the web area to a high degree of accuracy. Using this approach, we can more accurately calculate the relationship between web area and peripheral distance, thereby determining the degree of web compactness for individual spiders and their effective silk investment in constructing a web.

## Method details

### Field sampling of web data and initial image processing

Webs of *Seothyra schreineri* were located in a Nama Karoo habitat on the farm Bankfontein in the western Free State, South Africa. To create images, a steel ruler was placed next to each web to provide a scale, and a vertical photograph was taken using a Canon EOS 30D digital camera. Images were later imported into CorelDraw X7^TM^ and resized to correspond to the measurement tab (in millimetres). We then used the Bezier tool to trace the outline of each web, closed the curve, and examined the object properties to ascertain the peripheral length of the web. As an example, web A10.1 had a periphery of 174.364 mm (Fig. 1).

### Subsequent calculation of web area

At its base, the method consists of two parts. Firstly, segmentation of the image into two distinct regions, the web and the background, using a seeded region growing technique [10]. Secondly, counting the number of pixels in the web segment of the image and subsequent multiplication of the number of such pixels with the area represented by each.

**Fig. 2:**
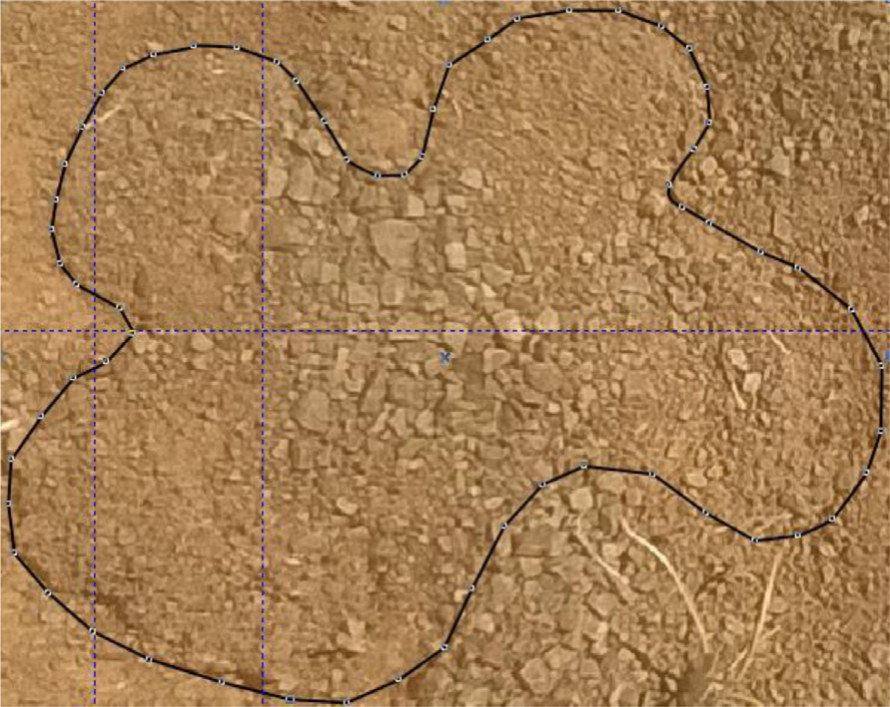
Cropped image of web A10.1, with the border outlined. Copyright © 2022 Charles Haddad, Corel Corporation and its licensors. All rights reserved.

Following the initial processing of spider web photographs, in each case a cropped image, with the web outlined, was prepared (Fig. 2).

All consequent transformations and calculations of web areas were completed in GNU Octave 7.1.0. The following details the stepwise process that was followed to calculate the web area:

1. First, it was necessary to threshold the original image to create a black-and-white image, with the web's boundary seen as white dots on a black background.
2. We then filled the gaps left in the boundary after the thresholding. This was achieved by drawing a white line between each boundary pixel and its closest boundary neighbours (Fig. 3).

**Fig. 3:**
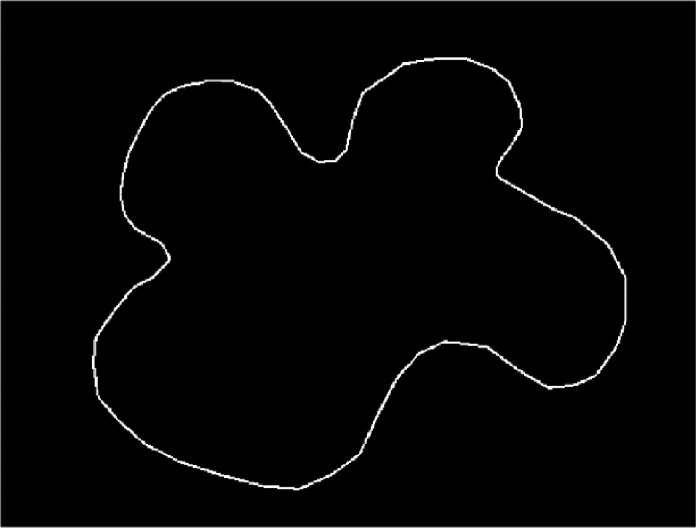
Image processed in GNU Octave 7.1.0: Web A10.1, with threshold applied and border gaps filled.
3. We then assigned the value of 1000 to a pixel in the middle of the image. This value was almost 4 times the maximum possible value (255) in the grayscale used by the images at that point, and thus made it easy to distinguish a pixel that had been identified as belonging to the web.
4. We considered pixels directly connected to a previously identified web-pixel to test if such pixels should also be classified as belonging to the web. Any pixel directly connected to at least one web-pixel, and which did not sit on the outside of the boundary, was given the value of 1000 to identify it as newly belonging to the web. This process can also be described in analogy to the spread of a virus within a geographically isolated population: A single pixel in the interior was infected (value of 1000). After many waves of transmission (Fig. 4), the virus kept on transmitting between pixels in contact with previously infected pixels until the boundary was found. The boundary was fully immunized, so could not get infected, and thus stopped the population of infected pixels from growing.

**Fig. 4:**
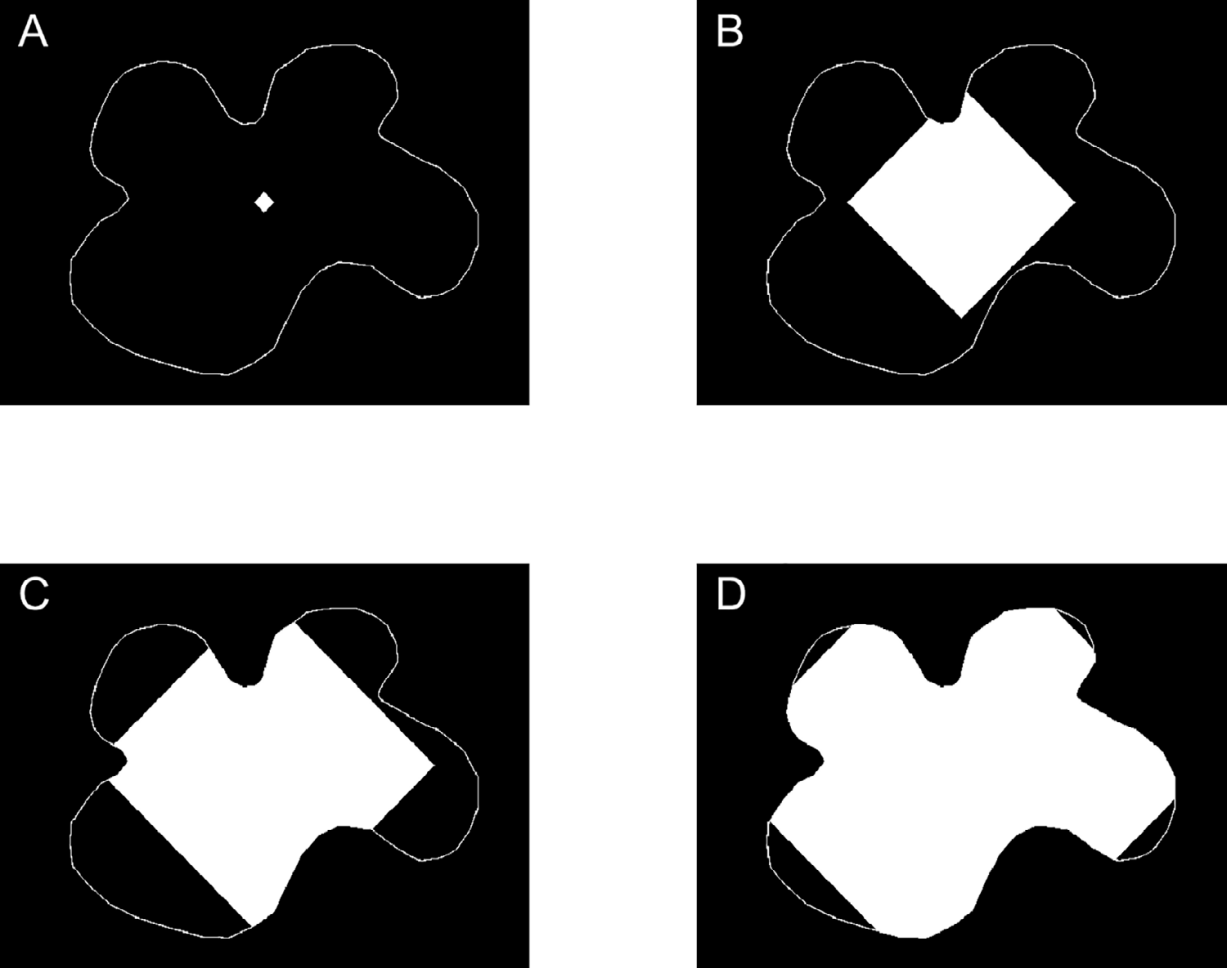
Region growing: Starting from a single web-pixel, the web-region after (**A**) 10, (**B**) 120, (**C**) 180 and (**D**) 260 iterations or waves of transmission.
5. We then calculated the area represented by one pixel by using the dimensions that were determined at the same time as the outlining of the boundary. As an example, for web A10.1 it was determined that the maximum width of the web was 52 mm, and the maximum height of the web was 41 mm. In Fig. 3, this width of 52 mm was covered by 430 pixels and the height of 41 mm was covered by 342 pixels. An area of 1 mm^2^ was thus covered by 69 pixels, on average.
6. The number of pixels with the value of 1000 (interior web-pixels) was then counted and added to the number of pixels with the value of 255 (the boundary pixels), and then added to calculate the web's area (Fig. 5) by dividing the number of web-pixels by the pixels/mm^2^. For web A10.1, the total number of web-pixels was 95838, and this then represented an area of 95838 ÷ 69 = 1389 mm^2^.

**Fig. 5:**
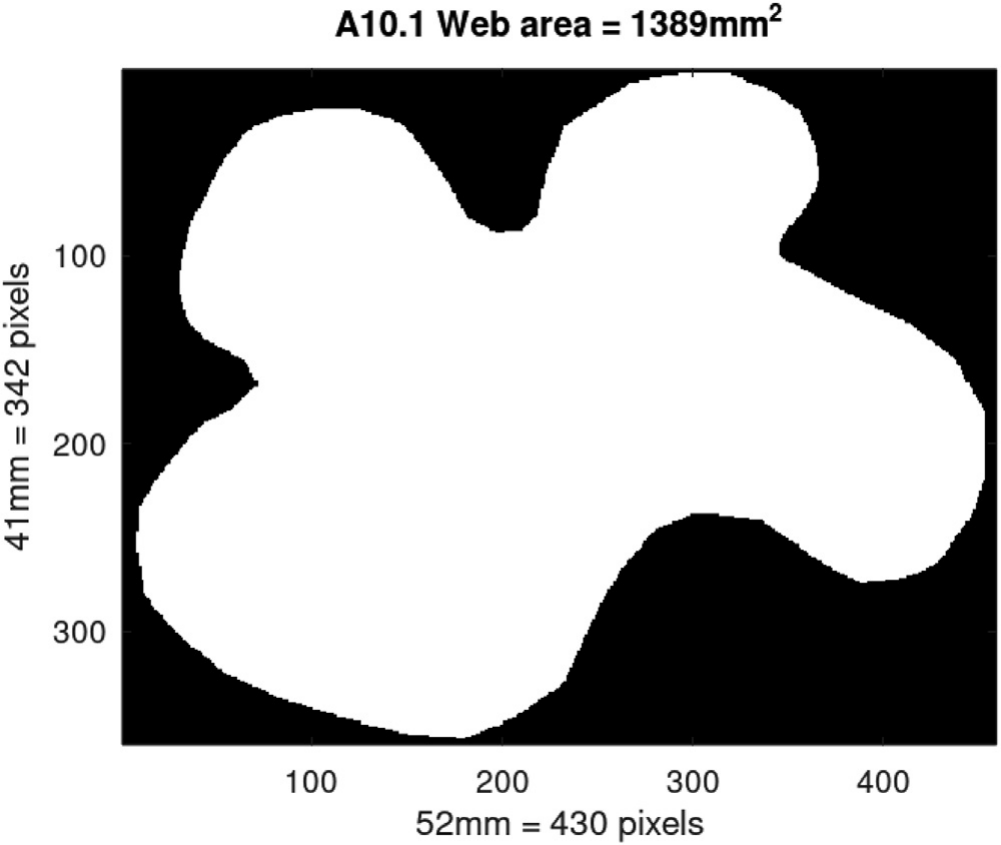
Final image of web A10.1 prepared in GNU Octave 7.1.0, with the web area determined as 1389 mm^2^.

### Reliability and validity of the method

**Fig. 6:**
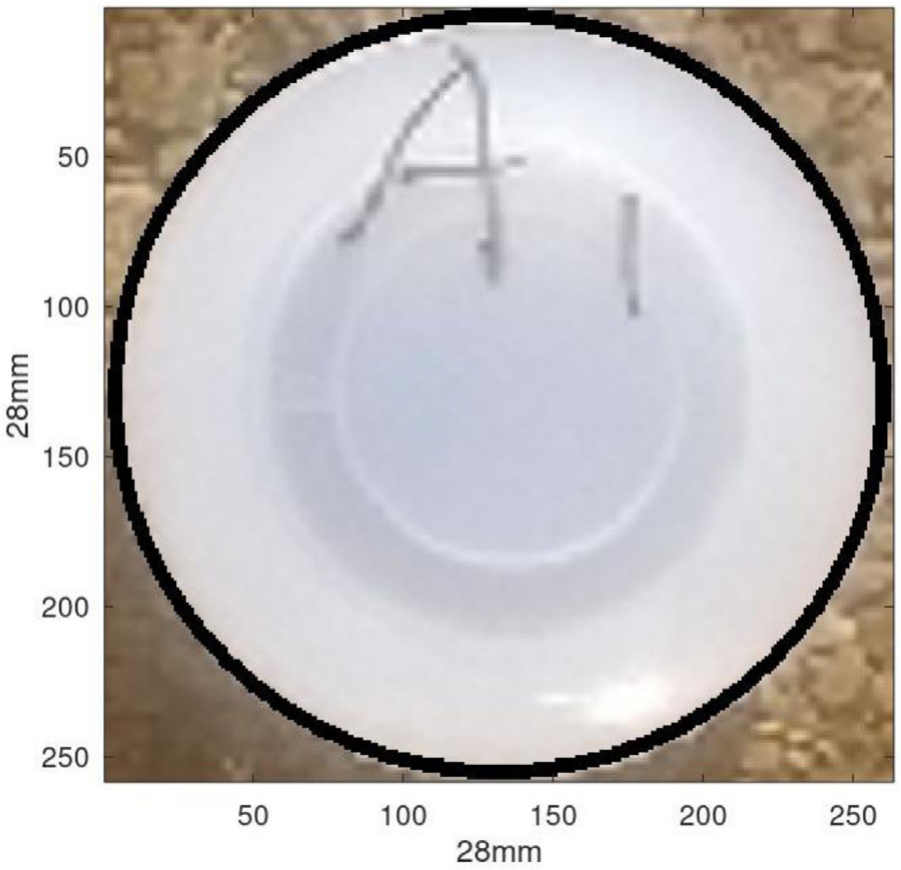
A specimen bottle lid with the diameter measured as 28 mm.

As a first attempt to verify the validity of the method, the above approach was used to calculate the area of the lid of a specimen bottle that had been placed close to the web. The bottle's top (Fig. 6) had a known diameter of 28 mm. Its area was thus easily calculated as:

$$A=\pi r^{2}=\pi {\left(14\right)}^{2}=616\;mm^{2}$$

Using the method described previously, the bottle top's area was determined to be 619 mm^2^. There was thus a difference of 0.5% between the actual and the determined area, which was deemed sufficiently small.

**Fig. 7:**
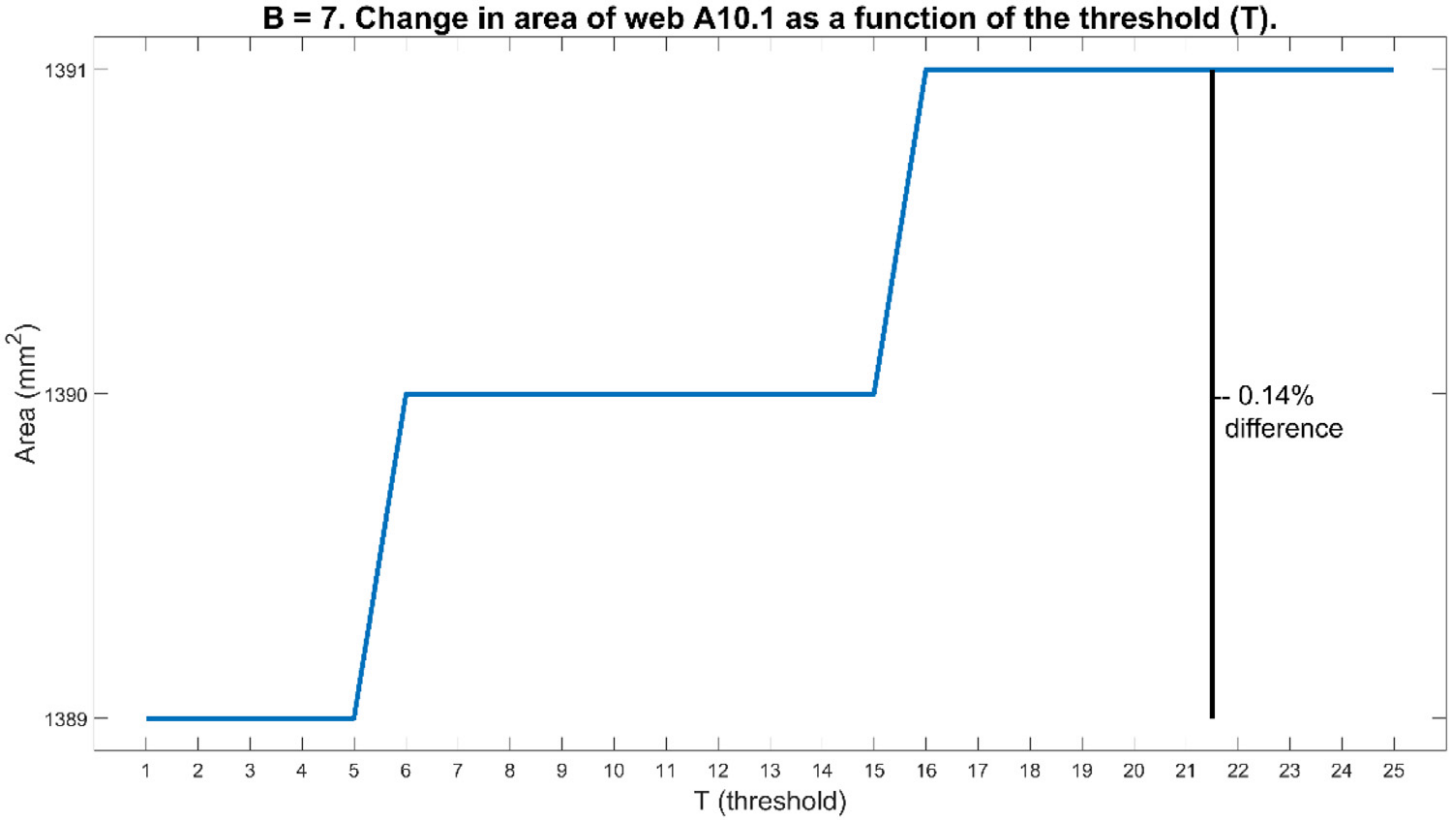
The area of web A10.1 varied between 1389 mm^2^ and 1391 mm^2^ with T∈[1,25] and B=7.

To investigate the reliability of the method, it was necessary to consider the parameter values used in the method. The first of these was the threshold value (T) used in step 1. The second was the number of neighbouring boundary pixels (B), used in step 2 to fill all gaps in the boundary. The method was found to be sensitive regarding the threshold value (T). Choosing T either too small or too large led to the method failing completely. The reason for failure was that such an erroneous T caused either too few boundary points to be found, or false boundary points to be found, which in both cases fundamentally changed the shape of the web's boundary. The valid interval for most images was T∈[1,25]. Changing the value of T to any value inside this interval did not change the determined areas by more than 0.5%. Fig. 7 shows, for web A10.1, how the calculated area varied when B was kept constant at B=7 and T varied from 1 to 25. For web A10.1 the maximum difference in area over this interval of T, was only 0.14%.

**Fig. 8:**
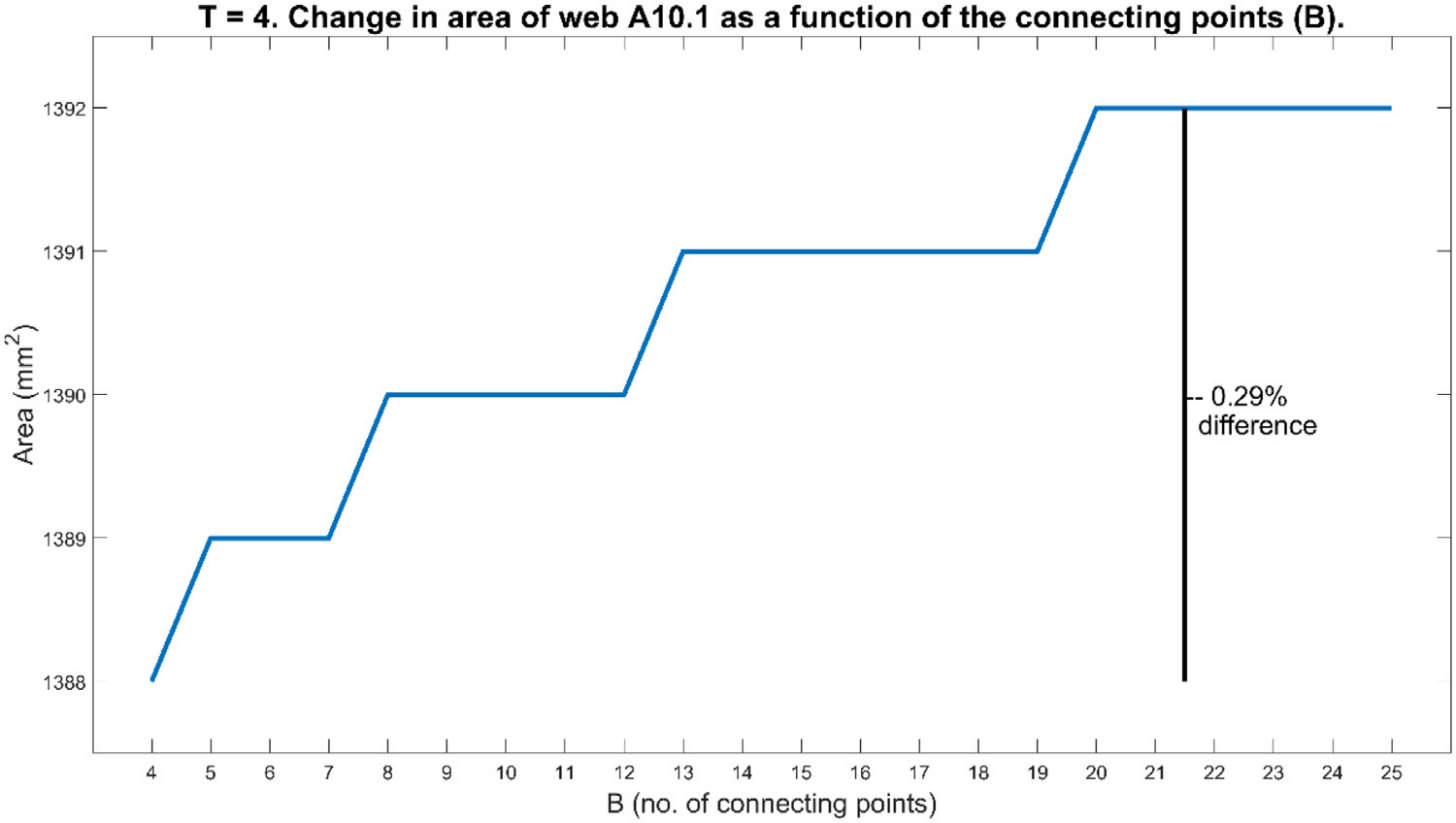
The area of web A10.1 varied between 1388 mm^2^ and 1392 mm^2^ with B∈[4,25] and T=4.

The method was also sensitive to the value of B. Using too small a value caused the method to fail, as it led to some gaps being left in the boundary. If even one gap was left in the boundary, the process described in step 4 could not be stopped, and thus a very large number of pixels sitting outside the web's boundary would falsely be identified as belonging to the web. Fortunately, it was easy to visually identify such instances. Such occurrences were rectified by choosing a slightly larger value for B to ensure all gaps in the boundary got filled. Care was taken to increase B as little as possible to ensure that the method worked, but not to increase B further than necessary, as this led to a false thickening of the boundary and thus falsely increasing the determined area. The smallest B that could ensure all boundary gaps were filled was used. Any small increase (less than 25%) in a working B-value did not cause more than a 0.5% change in the determined area, so reliability can still be concluded. Fig. 8 shows, for web A10.1, how the calculated area varied when T was kept constant at T=4 and B varied between 4 and 25. For web A10.1 the maximum difference in area over this interval of B was only 0.29%.

**Fig. 9:**
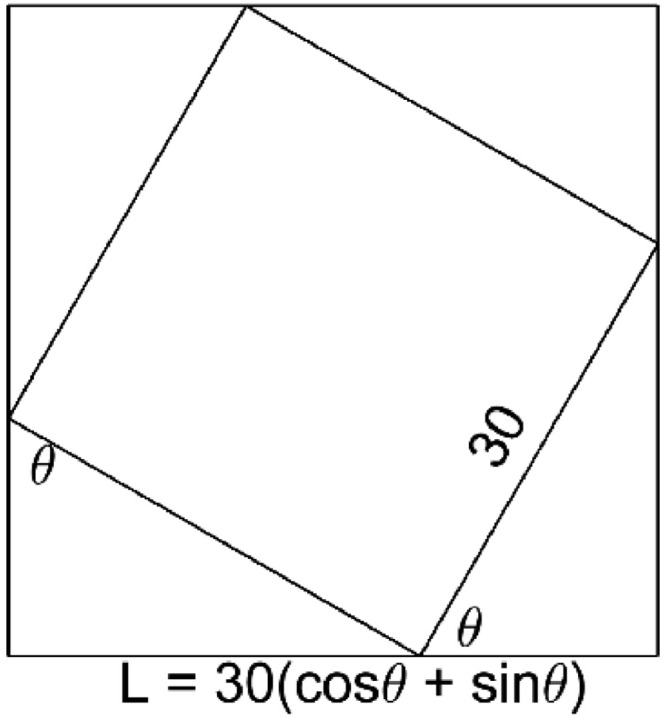
Square with a side length of 30mm, rotated $\theta^{\circ }$ anti-clockwise.

Further attempts to show the validity of the method were carried out. First, a square with a side length of 30mm was generated. To simulate what would happen if a photograph was taken at different rotations, we rotated the square at different angles and used our method to calculate the area in each case. The calculated area could then be compared with the known area of 30mm x 30mm = 900mm^2^. The calculation of area in our method needs the area represented by one pixel. To determine this, it is important to get the actual width (mm) and height (mm) of the shape. For our square being rotated, the width and height can be calculated in terms of the square's side length (30mm in this case) and the angle of rotation (θ) with respect to the horizontal. The bounding box/square (Fig. 9) then has a side length of L=30(cosθ+sinθ).

Table 1 shows the results of using our method to determine the areas of the original and rotated squares.

**Table 1:** Method used to determine the area of a square rotated at different angles.

As one more attempt to confirm validity, we used our method on some shapes less regular than a square. Graphs of four arbitrary functions were plotted and saved as images. Integration was used to determine the actual area of the shapes formed between the curves and the x-axis. The actual areas could then be compared to the areas determined by using our method on the images. For the examples, the functions were graphed and integrated over the interval [0,30]. Therefore, the width of each of the shapes was 30mm. The height of each shape was the maximum value of the function over the interval. Table 2 shows the results.

**Table 2:** Method used the determine the areas between four curves and the x-axis, 0≤x≤30.

From Tables 1 and 2 we see that the differences in actual vs determined areas using our method were relatively small. The maximum observed difference was 1.3%. The average difference was 0.71%. Whether a difference of 0.71% is considered significant, will depend on the context and the application.

## Conclusions

Here we proposed a new approach to determining the surface area of a two-dimensional irregular shaped, asymmetrical object, such as a buckspoor spider web, using a seeded region growing technique and pixel counting approach. The method was demonstrated to be highly accurate, so could potentially be applied to calculate the area of any two-dimensional shape. Further investigation could see the method adapted to determine volumes from photographs if aspects like thickness and a density profile is available.

A possible limitation to the current method concerns the need to know the dimensions of the shape quite accurately. When the area is determined, the pixel count will be highly accurate, however the accuracy of the result is dependent on knowing the actual width and height of the shape. If this cannot be determined after the fact, for example in the case of a spider web that might be altered or gone at some point, it is important to accurately measure or determine the dimensions of the shape in question when the photograph of the shape is taken.

## Declaration of Competing Interest

The authors declare that they have no known competing financial interests or personal relationships that could have appeared to influence the work reported in this paper.

## Data Availability

No data was used for the research described in the article.
